# A cross-sectional study of the individual, social, and built environmental correlates of pedometer-based physical activity among elementary school children

**DOI:** 10.1186/1479-5868-8-30

**Published:** 2011-04-12

**Authors:** Gavin R McCormack, Billie Giles-Corti, Anna Timperio, Georgina Wood, Karen Villanueva

**Affiliations:** 1 Population Health Intervention Research Centre, Department of Community Health Sciences, University of Calgary, Alberta, Canada; 2 Centre for Built Environment, School of Population Health, University of Western Australia, Australia; 3 Centre for Physical Activity and Nutrition Research, Deakin University, Melbourne, Victoria, Australia

**Keywords:** walkability, youth, obesity, socioeconomic status, environment

## Abstract

**Background:**

Children who participate in regular physical activity obtain health benefits. Preliminary pedometer-based cut-points representing sufficient levels of physical activity among youth have been established; however limited evidence regarding correlates of achieving these cut-points exists. The purpose of this study was to identify correlates of pedometer-based cut-points among elementary school-aged children.

**Method:**

A cross-section of children in grades 5-7 (10-12 years of age) were randomly selected from the most (n = 13) and least (n = 12) 'walkable' public elementary schools (Perth, Western Australia), stratified by socioeconomic status. Children (n = 1480; response rate = 56.6%) and parents (n = 1332; response rate = 88.8%) completed a survey, and steps were collected from children using pedometers. Pedometer data were categorized to reflect the sex-specific pedometer-based cut-points of ≥15000 steps/day for boys and ≥12000 steps/day for girls. Associations between socio-demographic characteristics, sedentary and active leisure-time behavior, independent mobility, active transportation and built environmental variables - collected from the child and parent surveys - and meeting pedometer-based cut-points were estimated (odds ratios: OR) using generalized estimating equations.

**Results:**

Overall 927 children participated in all components of the study and provided complete data. On average, children took 11407 ± 3136 steps/day (boys: 12270 ± 3350 vs. girls: 10681 ± 2745 steps/day; p < 0.001) and 25.9% (boys: 19.1 vs. girls: 31.6%; p < 0.001) achieved the pedometer-based cut-points.

After adjusting for all other variables and school clustering, meeting the pedometer-based cut-points was negatively associated (p < 0.05) with being male (OR = 0.42), parent self-reported number of different destinations in the neighborhood (OR 0.93), and a friend's (OR 0.62) or relative's (OR 0.44, boys only) house being at least a 10-minute walk from home. Achieving the pedometer-based cut-points was positively associated with participating in screen-time < 2 hours/day (OR 1.88), not being driven to school (OR 1.48), attending a school located in a high SES neighborhood (OR 1.33), the average number of steps among children within the respondent's grade (for each 500 step/day increase: OR 1.29), and living further than a 10-minute walk from a relative's house (OR 1.69, girls only).

**Conclusions:**

Comprehensive multi-level interventions that reduce screen-time, encourage active travel to/from school and foster a physically active classroom culture might encourage more physical activity among children.

## Background

For children and adolescents, participation in regular physical activity provides physical and mental health benefits [1]. Specifically, regular physical activity can protect against weight gain and adiposity among children [2,3], preventing the risk of adverse health outcomes including diabetes, hypertension, hyperlipidemia, depression, asthma, adverse orthopedic conditions and fatty-liver disease [4-6]. Several reviews have identified consistent correlates of physical activity among youth [7-10]. These correlates are multilevel - suggesting that a socio-ecological framework may best represent the determinants of physical activity among youth - and include biological (i.e., sex, ethnicity, age, parent weight status), psychological (i.e., self-efficacy, preferences, intention, attitude), behavioral (i.e., past activity, physical education, school sports), socio-cultural (i.e., parent activity and support, parent education, active siblings, friend support) and built environmental factors (i.e., access to facilities/programs, opportunities for exercise)[7,8]. Recent evidence shows there are associations between self-reported and objectively-assessed built environmental attributes and physical activity among children, including access to public recreational facilities (i.e., playgrounds/parks), transport infrastructure (i.e., sidewalks), and neighborhood safety and disorder (i.e., perceived safety and crime) [11,12].

To date, much of the research examining correlates of physical activity among youth have relied on self-report methods for capturing physical activity behavior, including parent proxy. However, self-reported physical activity is prone to memory and recall bias [13,14] and children's cognitive development may impair their ability to provide accurate recall [15]. Because they are relatively inexpensive and easy to use from both a study participant and research perspective, pedometers are increasingly being used to measure physical activity and as a motivational device in interventions designed to promote physical activity participation among children and adolescents [16,17]. Recently, various pedometer-based cut-points for children and adolescents have been suggested--primarily focusing on classification of those at risk of overweight and obesity [18-22]. These cut-points (i.e., 13000-16000 steps/day) are within the normal range of daily pedometer-based physical activity undertaken by youth [23]. For instance, preliminary research examining pedometer-based cut-points among a sample of Australian, Swedish and United States youth (6-12 years of age) suggested that boys who participated in ≥15000 steps/day and girls who participated in ≥12000 steps/day were less likely to be classified as overweight or obese compared with children who took fewer steps [19]. Higher pedometer-based cut-points, including ≥16000 steps/day for boys and ≥13000 steps/day for girls, have been reported where overweight was defined as a body fat percentage in the 85^th ^percentile [20] while lower pedometer-based cut-points among boys (≥13666 steps/day) and girls (≥9983 steps/day) 6-12 years of age have also been found [21].

Achieving any of the existing pedometer-based cut-points does not guarantee that a child will be healthy weight. Nevertheless, boys achieving ≥15000 steps/day and girls achieving ≥12000 steps/day for instance, are more likely to participate in at least 60-minutes of moderate-intensity physical activity per day [24] - noteworthy given evidence showing a negative association between physical activity and body composition [2,3,25]. Moreover, the pedometer-based cut-points of ≥15000 steps/day for boys and ≥12000 steps/day for girls have been used as a benchmark for assessing physical activity levels among youth in several countries including Australia [26,27] and Canada [28] - the results of which indicate that over half of all children and adolescents are not achieving these levels. Yet, little is known about factors that may encourage or inhibit children achieving this level of pedometer-based physical activity.

Identifying factors that increase or decrease the likelihood of participating in or above these suggested pedometer-based cut-points may lead to more effective interventions designed to promote physical activity among children and adolescents. The limited evidence regarding correlates of achieving pedometer-based cut-points suggests that sex, day of the week, age, participation in outside play, attending sports clubs, weather conditions, SES, participation in active transport and ethnicity may be important [29-33]. Nevertheless, other characteristics associated with meeting these recommendations may also exist, for instance, objectively or self-reported measures of the built and social environments.

Therefore the aim of this exploratory study is to extend the evidence-base by examining associations between socio-demographic characteristics, sedentary and active leisure-time behavior, independent mobility, active transportation and the built environment, and achievement of sex-specific pedometer-based cut-points [19], among Australian elementary school-aged children. Given the differential association between sex and achievement of the pedometer-based cut-points, the moderating effect of sex on the association between all other correlates and meeting this suggested benchmark will also be examined.

## Method

This current study forms part of the TRavel, Environment, and Kids project (TREK). The overall aim of this project was to examine the extent to which the urban design of neighborhoods supports or discourages active transportation among children in grades 5-7 attending public elementary schools in Perth, Western Australia. The research methodology for TREK is presented elsewhere [34], and is briefly described here. Parent and child written informed consent was obtained for all participants. The University of Western Australia Human Research Ethics Committee granted ethics approval for this study.

### Sampling and recruitment

Public elementary schools (n = 238) across metropolitan Perth were assigned a school-specific walkability index and socioeconomic status (SES) score[34]. The walkability index reflected the street connectivity and traffic exposure within a 2 km walkable service area (defined using the street and informal pedestrian network). Connectivity of the walkable service area was measured by a Pedshed - a ratio of the pedestrian network area to the maximum possible area within a two kilometer Euclidian radius around the school. Vehicular traffic exposure comprised the sum of kilometers of each road type (including primary distributors (≥15000 vehicles/day), district distributors (≥6000 vehicles/day), and local distributors (<6000 vehicles/day) divided by kilometers of access roads (<3000 vehicles/day)) within the walkable catchment area. School SES was based on the WA Department for Education and Training's 2001 Socioeconomic Index. Schools within each SES tertile (low, medium, and high) were ranked according to their walkbility score and the four top and bottom ranking schools within each tertile were invited to participate. Schools set in semi-rural locations and schools classified as 'high walkable' but located on a primary distributor road were excluded. Declining schools were replaced with the next ranked school. One additional high walkable/low SES school was recruited, due to the smaller number of children participating in this category. Overall, 69.4% of schools approached agreed to participate (n = 25). One class from each grade 5-7 in each school was randomly selected until a minimum of 30 children per grade was recruited. Overall, 1480 children (56.6% of all invited students) completed surveys, of those 1291 participated in the pedometer data collection and 1314 of their consenting parents also completed a self-administered survey.

### Data collection

Data were collected July-December 2007 using a child questionnaire, parent questionnaire and pedometers in addition to other measures not reported here. Items were assessed for test-retest reliability between March and May 2007 and where available these estimates have been presented here as kappa (κ) or intraclass correlation (ICC) coefficients.

#### Pedometer-assessed physical activity

Accusplit (AH120 M8) pedometers were used to record children's step counts. A similar model of pedometer was recently used to assess recommendations regarding moderate-intensity walking cadence [35]. The pedometers were worn level with the hip bone in line with the midpoint of the right knee. Children were asked to wear the pedometers at all times, except during water activities and while sleeping, for seven consecutive days including weekdays and weekend days. Children recorded whether they had worn the pedometer according to protocol each morning at school under the instruction of the classroom teacher. The in-built memory function negated the need for children to press reset each day and manually record step counts. After seven days, the pedometers were collected and the number of steps double punched by a data entry clerk to minimize data entry error.

#### Socio-demographic variables

Children reported whether they had friends in the neighborhood (i.e., many vs. few friends) while parents reported whether their household had a dog (i.e., owner vs. non-owner). The child's sex and grade were also collected in the children's questionnaire, and highest education for any parent (i.e., at least one parent in household with: high school or less, completed diploma/college/technical school, or university education), marital status (i.e., married/defacto vs. other), the number of dependents <18 years at home (i.e., 1 child vs. ≥2 children), and home ownership (i.e., renting vs. owned/purchasing) were assessed in the parent questionnaire.

#### Sedentary and active leisure-time behavior

Parents reported the average amount of time per day their child spent using a computer or internet for pleasure, watching television/videos, and playing passive or active electronic games. Minutes of each activity were summed and dichotomized to reflect current screen-based activity recommendations for Australian youth (i.e., ≤2 vs. >2 hours/day)[36]. Children reported whether they had participated in the following leisure activities: playing in a park, playground, or playing field; playing team sport; attending a club or youth group; going for a walk in the neighborhood; playing in the street; playing in the yard; and taking the dog for a walk in the last week. An active leisure index was estimated representing the count of the different activities participated in by the child. To examine the possible effect of other students' physical activity levels on an individual child's physical activity behavior (i.e., peer influence), average pedometer steps within each grade within each school was also estimated.

#### Independent mobility and transportation to school

Parents were asked whether or not their child was allowed to play in the street (κ = 0.56), play at the closest park, playground, or playing field (κ = 0.55), or walk in their neighborhood (κ = 0.59) without an adult. A dichotomous variable was derived from these items - i.e., not allowed vs. allowed to play or walk in the neighborhood unsupervised. Parents also reported whether their child travelled to and from school by motor vehicle during a usual week (κ = 0.72).

#### Self-reported neighborhood environment

On a five-point Likert scale (i.e., strongly agree to strongly disagree) parents reported whether: they had to drive to get to a park with appealing equipment for their child; they often saw adults walking in their neighborhood; they often saw children walking in their neighborhood; their neighborhood was friendly; there was a lot of traffic in their neighborhood; the neighborhood was a nice place to walk around; there were safe crossings to reach a local shop; there were safe crossings to reach the closest local park; and drivers near the school often exceeded the speed limit (seven-day test-retest reliability n = 89 adults, ICC = 0.42-0.66). Parents also reported the time (i.e., <5, 5-10, 11-15, 16-20, or >20 minutes) required for their child to walk from home to their nearest: primary school; shop; newsagent; library; transit station; park; bushland; sports field; beach, and; river. The number of different types of destinations perceived to be within a 10-minute from home was counted. The presence of a relative and friend's house within a 10-minute walk of home were examined separately. Reported time to walk to these destinations had acceptable reliability (ICC = 0.51-0.84).

#### School neighborhood walkability and socioeconomic status

The school-specific walkability index, as previously described [34], was dichotomized into high vs. low walkable. School area socioeconomic status (SES) was collapsed into tertiles (i.e., high, medium, and low SES).

#### Data analysis

The analytical sample included cases who participated in all components of the study and had complete survey and valid pedometer data (n = 927). Pedometer-based physical activity was considered valid if counts were between 1000 and 30000 steps per day [37]--step counts outside this range were recoded as missing (5.9-32.0% for any given day). Average daily steps was calculated for participants recording steps for at least four days [38]. Moderate inter-day reliability was found for pedometer data measured for four or more days (ICC = 0.65). Average daily steps were categorized into sex-specific pedometer-based cut-points (<15000 vs. ≥15000 steps/day for boys and <12000 vs. ≥12000 steps/day for girls) [19]. These cut-points were chosen in part because of their use as a benchmark in population-based physical activity surveys in Western Australia [26] and elsewhere [28] and because the cut-points were established using an international sample which included Australian youth [19].

Descriptive statistics were computed for all characteristics. Moreover, characteristics of the analytical sample (n = 927) and excluded (n = 553; i.e., parent not participating in survey, child not participating in the pedometer data collection, or child not providing valid pedometer data) respondents were compared using Pearson's Chi-square and independent t-tests. To reduce the number of parent neighborhood environment perception variables (i.e., drive to get to a park with appealing equipment for their child; often seeing adults walk in their neighborhood; often seeing children walk in their neighborhood; neighborhood is friendly; a lot of traffic in their neighborhood; neighborhood a nice place to walk around; safe crossings to reach a local shop; safe crossings to reach the closest local park, and; drivers near the school often exceeded the speed limit) a principal component analysis was performed. Items belonging to the same construct were identified (i.e., varimax rotated loadings >0.40 and on a single factor) and summed. Two factors were identified, including: 1) neighborhood friendliness (n = 4 items; explained variance = 30.9%; factor loadings = 0.70-0.76; Cronbach's alpha = 0.74); and 2) traffic barriers (n = 4 items; explained variance = 19.3%; factor loadings = 0.58-0.78; Cronbach's alpha = 0.66). A single item--drive to get to a park with appealing equipment for their child--did not load on either factor and was examined separately in the analysis.

To account for school-level clustering, generalized estimating equations (GEE) were used (i.e., using an exchangeable correlation matrix and robust standard errors estimated) to estimate odds ratios (OR) and 95 percent confidence intervals (95%CI). Using GEE, achievement of the pedometer-based cut-points was regressed onto the built environment, social environment, sedentary behavior, active leisure-time behavior, independent mobility, travel to school, and peer pedometer-based physical activity. To assess the moderating effect of sex, interaction terms between sex and the other correlates were estimated and a backward stepwise removal of non-significant interaction terms (p > 0.05) undertaken to derive the final model. All main effects were retained in the model regardless of statistical significance. Based on bivariate correlations (r < 0.35) and variance inflation factors (VIFs < 1.4) among the correlates, the level of multicollinearity within the regression model was not an issue. Statistical analysis was undertaken using Predictive Analytic Software 17 (SPSS, Inc).

## Results

Compared with the excluded study participants, the analytical sample included a significantly higher proportion of girls, those spending <2 hours on screen-based activity/day, those allowed to play in the neighborhood without adult supervision, and those attending a school in a higher SES neighborhood (Table 1). Moreover, the analytical sample took more daily pedometer steps (11407 ± 3136 vs. 10294 ± 3891 steps/day, p < 0.001), were from grades that took a higher number of pedometer steps/day (11172 ± 1231 vs. 10846 ± 1398, p < 0.001), participated in more leisure physical activities (3.63 ± 1.58 vs. 3.45 ± 1.66, p = 0.013), and had parents who reported a higher mix of destinations within a 10-minute walk of home (5.45 ± 2.48 vs. 3.65 ± 3.24, p < 0.001) compared with excluded participants (not shown in the Table). Parent perceived neighborhood traffic and friendliness did not significantly differ between the analytical and excluded samples (traffic: 2.90 ± 0.78 vs. 2.85 ± 0.77 and friendliness: 3.95 ± 0.61 vs. 3.90 ± 0.63, respectively). Excluding outliers, the minimum and maximum average daily steps for the analytical sample were 3164 and 22878 steps/day, respectively. Although boys on average took more daily steps than girls (boys: 12270 ± 3350 vs. girls: 10681 ± 2745 steps/day; p < 0.001), fewer boys achieved the pedometer-based cut-points (boys: 19.1 vs. girls: 31.6%; p < 0.001).

**Table 1:** Comparison of child and parent-reported questionnaire data and pedometer data among the analytical sample and cases excluded in the current study

		Excluded cases (n = 553)	Analytical sample (n = 927)	
**Variables**		**%**	**%**	***p***

Home ownership*	Renting	28.8	23.9	0.084
	Owned/purchasing	71.2	76.1	
Marital status*	Married/defacto	80.5	78.2	0.370
	Other	19.5	21.8	
Dependents at home <18 yrs*	One child	10.7	12.6	0.370
	≥2 children	89.3	87.4	
Sex	Male	53.7	45.7	0.003
	Female	46.3	54.3	
Grade of student	Grade 5	25.1	27.3	0.211
	Grade 6	34.4	36.8	
	Grade 7	40.5	35.9	
Highest education completed by any parent*	≤High school	25.5	23.4	0.266
	Diploma/trade/other	53.2	51.1	
	≥Bachelor degree	21.3	25.5	
Child usually driven to and from school	No	40.1	35.8	0.147
	Yes	59.9	64.2	
Screen-based activity time*	<2 hours/day	18.1	27.5	<0.001
	≥2 hours/day	81.9	72.5	
Dog ownership *	Owner	55.2	57.1	0.547
	Non-owner	44.8	42.9	
Child has friends in the neighborhood	Many friends	69.5	68.7	0.762
	Few friends	30.5	31.3	
Distance to nearest friends house from home*	>10 minute walk	35.0	38.6	0.226
	≤10 minute walk	65.0	61.4	
Distance to nearest relatives house from home*	>10 minute walk	83.8	83.2	0.773
	≤10 minute walk	16.2	16.8	
Have to drive child to park with suitable equipment	Yes	39.9	35.9	0.179
	No	60.1	64.1	
Play in any street, park, or go for walk without an adult*	Not allowed	20.2	14.0	0.006
	Allowed	79.8	86.0	
School neighborhood walkability score	High	47.7	45.6	0.431
	Low	52.3	54.4	
School neighborhood socioeconomic status	High	32.4	41.2	<0.001
	Medium	33.1	35.8	
	Low	34.5	23.0	
Pedometer-based physical activity	Not achieving suggested steps	79.5	74.1	0.058
	Achieving suggested steps	20.5	25.9	

* Indicates data collected from parent questionnaire; pedometer-based cut-points: ≥12000 steps/day for girls and ≥15000 steps/day for boys

N = 1480 participated in child survey; N = 1314 participated in parent survey; N = 1291 children participated in pedometer data collection.

Note: Excluded cases were those not participating in all components of the study as well as those not providing complete or valid data

### Correlates of pedometer-based cut-points

The estimated exchangeable working correlation matrix suggested clustering of achievement of pedometer determined physical activity recommendations among students within the same school (r = 0.018; number of respondents per grade: minimum = 7; maximum = 65). Seven variables were associated (p < 0.05) with achieving the suggested pedometer-based cut-points (female, < 2 hours/day screen-based activity, not being driven to school, the average pedometer steps among other students within the child's school grade, the mix of neighborhood destinations perceived to be within 10 minutes from home, proximity of a friend's house, and higher school neighborhood SES) (Table 2). After adjustment, compared with girls, boys had lower odds of achieving the pedometer-based cut-points (OR 0.42). Participants had higher odds of achieving the pedometer-based cut-points if they participated in less than two hours of screen-based activity/day (OR 1.88) or if they were not usually driven to and from school (OR 1.48). Moreover, the average number of steps among children within the respondent's grade was positively associated with achieving the pedometer-based cut-points. For each 500 steps/day taken by students belonging to a child's grade at the same school, the odds of achieving the cut-points increased by 29% (OR 1.29) (Table 2). Noteworthy, was that the odds of achieving the pedometer-based cut-points decreased as the parent perceived more destinations to be present within a 10-minute walk from home (OR 0.93). Moreover, children who resided more than 10-minutes from their friend's house had lower odds of achieving the pedometer-based cut-points (OR 0.62), while attending a school located in a high SES neighborhood was positively associated with achieving the cut-points (OR 1.33). Notably, the overall walkability of the school walkable service area was not significantly associated with achieving the suggested pedometer-based cut-points.

**Table 2:** Odds ratios (OR) and 95% confidence intervals (95%CI) for the associations between socio-demographic, built environmental, and behavioral correlates of achieving pedometer-based cut-points

			Main effects		Main effects + interactions	
**Variables**		**%**	**OR**	**95%CI**	**OR**	**95%CI**

**Socio-demographic**						
Home ownership*	Renting	25.7	1.11	0.80, 1.54	1.11	0.80, 1.56
	Owned/purchasing	26.0	Ref.		Ref.	
Marital status*	Married/defacto	25.5	1.00	0.59, 1.69	0.98	0.58, 1.66
	Other	27.2	Ref.		Ref.	
Dependents at home <18 yrs*	One child	29.9	1.39	0.87, 2.21	1.42	0.89, 2.26
	≥2 children	25.3	Ref.		Ref.	
Sex	Male	19.1	0.42	0.31, 0.58^Φ^	0.03	0.01, 0.79^Φ^
	Female	31.6	Ref.		Ref.	
Grade of student	Grade 5	28.1	1.12	0.81, 1.54	1.12	0.81, 1.55
	Grade 6	26.1	0.86	0.63, 1.18	0.90	0.65, 1.25
	Grade 7	24.0	Ref.		Ref.	
Highest education completed by any parent*	≤High school	24.0	1.25	0.73, 2.13	1.28	0.77, 2.13
	Diploma/trade/other	26.2	1.36	0.94, 1.97	1.38	0.95, 2.03
	≥Bachelor degree	27.1	Ref.		Ref.	
Dog ownership*	Owner	26.1	1.08	0.75, 1.55	1.07	0.74, 1.53
	Non-owner	25.6	Ref.		Ref.	
Child has friends in the neighborhood	Many friends	27.8	1.04	0.73, 1.49	1.02	0.70, 1.47
	Few friends	21.7	Ref.		Ref.	
**Sedentary and active leisure-time behavior**						
Screen-based activity time*	<2 hours/day	35.7	1.88	1.30, 2.73^Φ^	1.89	1.29, 2.78^Φ^
	≥2 hours/day	22.2	Ref.		Ref.	
Leisure physical activity index [min. = 0, max. = 7]^			1.08	0.95, 1.36	1.08	0.95, 1.22
Average steps within grade (500 step increments) [min. = 7779.31, max. = 14588.97]^			1.29	1.22, 1.36^Φ^	1.21	1.13, 1.31^Φ^
**Independent mobility and transportation to school**						
Play in any street, park, or go for walk without an adult*	No	22.3	0.71	0.44, 1.14	0.73	0.45, 1.18
	Yes	26.5	Ref.		Ref.	
Child usually driven to and from school	No	21.0	1.48	1.08, 2.04^Φ^	1.44	1.04, 2.00^Φ^
	Yes	33.0	Ref.		Ref.	
**Self-reported neighborhood environment**						
Have to drive child to park with suitable equipment	Yes	27.1	0.99	0.72, 1.35	0.98	0.72, 1.33
	No	23.7	Ref.			
Traffic in neighborhood [min. = 1, max. = 5]*			0.96	0.80, 1.15	0.97	0.81, 1.16
Friendliness of neighborhood [min. = 1, max. = 5]*			1.15	0.94, 1.41	1.18	0.94, 1.47
Distance to nearest friends house from home*	>10 minute walk	20.4	0.62	0.47, 0.82^Φ^	0.60	0.46, 0.78^Φ^
	≤10 minute walk	29.3	Ref.		Ref.	
Distance to nearest relatives house from home*	>10 minute walk	26.1	1.05	0.61, 1.80	1.66	0.97, 2.85
	≤10 minute walk	25.0	Ref.		Ref.	
Count of different destinations within a 10 minute walk of home [min. = 0, max. = 13]*			0.93	0.88, 0.99^Φ^	0.93	0.88, 0.99^Φ^
**Neighborhood walkability and socioeconomic status**						
School neighborhood walkability score	High	28.6	1.01	0.75, 1.35	1.00	0.74, 1.35
	Low	23.6	Ref.		Ref.	
School neighborhood socioeconomic status	High	29.6	1.33	1.06, 1.67^Φ^	1.36	1.11, 1.68^Φ^
	Medium	25.0	1.15	0.82, 1.60	1.15	0.82, 1.60
	Low	20.7	Ref.		Ref.	
**Sex-specific interactions**						
Sex by dist. relatives house within 10-min walk					0.27	0.15, 0.47^Φ^
Sex by average steps within grade					1.17	1.02, 1.34^Φ^

^Φ ^p < 0.05. Ref.: reference category. * Indicates data collected from parent questionnaire; Pedometer-based cut-points: ≥12000 steps/day for girls and ≥15000 steps/day for boys. ^ Parameter estimates represent linear associations between the variable and pedometer-based physical activity. All estimates adjusted for school-level clustering

The child's sex was found to moderate associations between distance to the closest relative's home and the average daily steps among all students within the respondent's grade at their school and achievement of the pedometer-based cut-points (Table 2). Sex-stratified models revealed that boys had lower odds (OR 0.44), while girls had higher odds (OR 1.69), of achieving the pedometer-based cut-points if they resided further than a 10-minute walk from a relative's home. Moreover, the association between the average pedometer-based physical activity among students within the respondent's grade and achievement of the pedometer-based cut-points was slightly higher for boys (OR 1.43) than for girls (OR 1.23) (Table 3).

**Table 3:** Sex-stratified models showing odds ratios (OR)* and 95% confidence intervals (95%CI) of the association between average steps within the respondent's grade, distance to nearest relatives house from home, and achievement of pedometer-based cut-points

		Achieving the pedometer-based cut-points			
		**Boys (n = 424)**		**Girls (n = 503)**	

**Variables**		**OR**	**95%CI**	**OR**	**95%CI**

Average steps within grade (in 500 step increments)		1.43	1.27, 1.60^Φ^	1.23	1.14, 1.33^Φ^
Distance to nearest relatives house from home*	>10 minute walk	0.44	0.22, 0.86 ^Φ^	1.69	0.98, 2.90
	≤10 minute walk	Ref.			

* Adjusted for all built environmental, social environmental, socioeconomic, demographic, and behavioral correlates presented in Table 2

^Φ ^p < 0.05. Ref.: reference category

## Discussion

This study provides new evidence adding to the existing literature on correlates of pedometer-based physical activity in children. We found that the achievement of pedometer-based cut-points [19] was associated with the child's own behavior (i.e., active transport to school, screen-based activity), the physical activity levels of their peers, and the characteristics of their neighborhood (i.e., school neighborhood-SES, proximity of a friend's house, the presence of local destinations).

Girls were more likely to achieve the suggested pedometer-based cut-points. Consistent with previous evidence [27-29,33] boys accumulated more daily steps than girls, yet they were less likely than girls to achieve this level of pedometer-based physical activity. Duncan et al. [39] found a similar pattern among 8-11 year old boys and girls. This discordance likely reflects the higher pedometer-based cut-point for boys (≥15000 steps/day) compared with girls (≥12000 steps). Only nineteen percent of boys and 32% of girls achieved the pedometer-based cut-point - somewhat lower than the prevalence for elementary school-aged children (grade 3, 5, and 7) found in a recent Western Australian survey (boys = 31.7% and girls = 43.9%) [26]. However, the studies differed in a number of ways, including the specific grades captured, the sample design, the season in which data were collected, as well as the method for collecting and recording pedometer data (i.e., steps recorded daily vs. steps recorded after seven-days from the in-built memory). Nevertheless, our results correspond more closely with recent Australian national data suggesting that 24% of boys and 33% of girls ages 9-13 achieved these same pedometer-based cut-points [27]. We found no association between school grade and achievement of the pedometer-based cut-points, consistent with studies that have found no relationship between steps and age among children [22,39].

Previous research has shown positive associations between maternal education, family income and physical activity levels among adolescents however, the relationship between SES and physical activity among children is less clear [8]. We found no association between household SES--parent education and home ownership--and achievement of the pedometer-based cut-points. Children attending a school in a higher socioeconomic neighborhood were more likely to achieve the pedometer-based cut-points compared with those attending schools in low socioeconomic neighborhoods, even after accounting for household indicators of SES. Disadvantaged neighborhoods often offer fewer physical activity opportunities for children [40]. While our walkability index captured pedestrian connectivity and traffic exposure, it is possible that other environmental characteristics related to neighborhood SES exist (e.g., land-use mix, aesthetics, and safety).

Level of independent mobility, such as going to local shops, school, friends, or park unsupervised, may be associated with increased outdoor play, participation in structured sport and exercise, and walking and cycling to and from school [41,42]. As well as potentially decreasing physical activity opportunities, restricting independent mobility may negatively influence a child's cognitive and social development by reducing opportunities to socialize and explore and interact with their neighborhood environment [43,44]. Moreover, children with restricted mobility may spend more time participating in passive modes of transportation, particularly for travel to school [45]. Although children who were not allowed to play in their neighborhood unsupervised were less likely to achieve the pedometer-based cut-points, this was not statistically significant. Nevertheless, we did find that children who were usually driven to or from school were less likely to achieve the pedometer-based cut-point, suggesting that active transportation such as walking can contribute to overall levels of physical activity. Positive associations between levels of active transportation and physical activity, including pedometer-based physical activity among youth have been reported elsewhere [46,47]. Creating safe child-friendly neighborhoods, changing parent and child perceptions and attitudes towards motor vehicle use, encouraging parents to walk or be active in their neighborhoods with their children, and implementing sustainable walk to school programs could encourage independent mobility and active transportation which may contribute to increasing levels of physical activity.

There has been a recent focus on the independent contribution of sedentary behavior on the health of both adults and children [48,49]. We found that children who spent more than two hours daily on screen-based activity took fewer steps and were less likely to achieve the pedometer-based cut-points. Our finding is important as only modest or null associations between time spent in screen-based activity and steps among children have been previously found [49-51]. Interventions that decrease screen-based activity could contribute to higher levels of physical activity participation among children.

School neighborhood walkability and parent perceptions of traffic barriers and safety or neighborhood friendliness were not associated with pedometer-based physical activity. We did find, however, a negative relationship between the count of different neighborhood destinations that parents perceive to be within a 10-minute walk of home and achieving the pedometer-based cut-points. Some destinations captured in this study might be considered less important for encouraging children to be more physically active (e.g., libraries and news agencies). The presence of more local destinations might also result in the neighborhood being less supportive of physical activity among children of this age because of more destination-related motor vehicle traffic, limited area for play spaces and the presence of more strangers visiting local neighborhoods. An alternative explanation is that fewer steps are needed to travel to destinations that are closer to home. Nevertheless, Frank et al. [52], for example, found objectively-assessed mix of land uses to be positively associated with walking among adolescents but not children aged 5-11 years. Furthermore, others have noted negative associations between environmental characteristics usually supportive of physical activity among adults (i.e., street connectivity) and walking in children [53]. The lack of association between the perceived neighborhood environment and pedometer-based physical activity is not surprising as evidence of this relationship among pre-adolescent children is mixed [54].

Children whose friend's house was within a 10-minute walk were more likely to achieve pedometer-based cut-points. Children with friends residing nearby might visit more frequently using active modes, may be involved in active play, and may have company to walk to and from school. Together this may result in more walking and physical activity. There is evidence for associations between friend support and physical activity among children [8]. In support, we also found a positive association between a child's pedometer-based physical activity and the physical activity level of their peers (within the same grade level at their school) - with the association slightly stronger for boys than girls. Peer physical activity levels might encourage physical activity behaviors among youth [55]. Our finding could reflect peer modeling or support, or unmeasured influences of the school (i.e., quantity and quality of physical education or school sports) or the neighborhood environment on physical activity. In addition, boys were less likely to achieve the pedometer-based cut-point if they resided further than a 10-minute walk of their nearest relative's home, whereas girls were more likely to achieve the cut-point. Speculatively, girls might be more willing to walk further than boys to visit relatives resulting in higher recorded pedometer-based physical activity. More research is needed to disentangle these associations.

Several limitations should be considered when interpreting the findings of this study. Differences between the analytical sample and the excluded participants were observed. In particular the analytical sample was more physically active, thus the results presented might underestimate the magnitude of the associations between the correlates and pedometer-based physical activity. Moreover, the moderate reliability of some variables may have attenuated the estimated associations between these correlates and the outcome. The generalizability of these results are limited to school-aged children in grades 5-7 attending elementary schools in high and low walkable neighborhoods determined based on pedestrian connectivity and traffic exposure. Other objectively-assessed environmental characteristics not measured here could be important for determining pedometer steps among children, although more research is needed to identify these characteristics. We examined the correlates of one set of recently suggested pedometer-based physical activity cut-points [19], thus the associations found here may not generalize to other existing pedometer-based cut-points for children and adolescents. Children and adolescents achieving the pedometer-based cut-points used in this study may be less likely to be classified as overweight and obese compared with those not achieving these cut-points [19] however, we did not examine the association between steps and weight status or any other health outcome. Future research should investigate the extent to which achievement of the various established pedometer-based physical activity cut-points result in positive health outcomes including, but not limited to, improved weight status among children and adolescents. Finally, associations found from this cross-sectional study cannot be considered causal.

## Conclusions

For children, participation in regular physical activity can provide physical and mental health benefits and physical activity behaviors adopted during childhood often track into adulthood [56]. These results suggest that children, who participate in less screen-based activities at home, use active modes to travel to and from school and attend schools that foster a physically active classroom culture take more pedometer-based steps daily. Multi-level interventions, programs, and policies are required particularly targeting lower socioeconomic schools. More research examining correlates of pedometer-based physical activity separately for boys and girls is needed.

## Abbreviations

(BMI): body mass index; (ICC): intraclass correlation; (OR): odds ratio; (SES): socioeconomic status; (95%CI): 95 percent confidence interval;

## Competing interests

The authors declare that they have no competing interests.

## Authors' contributions

BGC, AT, and GRM conceived this study. GW and KW contributed to data collection. GRM conducted the analysis. All authors contributed to interpretation and writing of the manuscript. All authors read and approved the final manuscript.
